# Role of aquaporins in brain water transport and edema

**DOI:** 10.3389/fnins.2025.1518967

**Published:** 2025-01-29

**Authors:** Yuyuan Li, Yining Wang, Xingda Huang, Hao Zhang, Youfei Guan, Xiaoyan Zhang

**Affiliations:** ^1^Advanced Institute for Medical Sciences, Dalian Medical University, Dalian, China; ^2^Health Science Center, East China Normal University, Shanghai, China

**Keywords:** aquaporins, water channels, water transport, brain edema, cytotoxic, vasogenic, hydrocephalus

## Abstract

Water serves as the primary substance in all living cells and is an essential molecule involved in numerous biological processes critical for maintaining homeostasis in the central nervous system (CNS). Disruptions in water balance can occur in conditions such as cerebral edema, where fluid accumulation results in increased intracranial pressure (ICP). Aquaporins (AQPs) are transmembrane proteins that play a vital role in the rapid transport of water across cell membranes. Various subtypes of AQPs (AQP1, AQP3, AQP4, AQP5, AQP6, AQP7, AQP8, AQP9, and AQP11) have been identified in brain tissue. This review summarizes the latest advancements in our understanding of the critical role of AQPs in regulating water transport in brain edema. Abundant evidence indicates that AQP4, the most prevalent AQP in the CNS, regulates brain water transport and contributes to both cytotoxic and vasogenic edema, suggesting that AQP4 may serve as a potential therapeutic target for brain edema. Additionally, some studies have indicated that AQP1 plays a significant role in the formation of cerebrospinal fluid (CSF) and the maintenance of steady-state ICP. However, to date, these findings have not been translated into clinical practice. There is an urgent need to develop specific AQP inhibitors and activators to explore the potential benefits of modulating the functions of AQP1 and AQP4 in the context of brain edema.

## 1 Introduction

Water is the most abundant component of all living cells and organisms, and the effective regulation of water balance is crucial for numerous biochemical processes. Approximately 80% of the brain is composed of water, which circulates among various compartments, including blood, cerebrospinal fluid (CSF), and the intracellular and interstitial spaces of brain parenchyma. It traverses the blood-brain and the blood-CSF interfaces. Disturbances in water homeostasis can have severe detrimental effects on brain function and significantly contribute to the pathophysiology of traumatic brain injury (TBI), stroke, and various cerebral disorders (Fishman, 1975).

Cerebral edema is defined as the excessive accumulation of water within the brain parenchyma, which is associated with various cerebral injuries, including ischemic injury, TBI, and brain tumors. This condition ultimately leads to an increase in intracranial pressure (ICP) (Chen et al., 2024). Elevated ICP can further impair the regulation of cerebral blood flow (CBF), eventually resulting in additional brain injury and potentially death. The regulation of brain water balance is of clinically significant. However, the exact mechanisms involved in water accumulation and clearance in the brain remain unclear. Currently, the treatment of cerebral edema mainly focuses on removing excess water from the brain parenchyma and reducing ICP, which helps to limit the damage but fails to address the underlying causes of brain edema. Therefore, the development of novel therapeutic drugs that directly target the molecular key players in the mechanisms of edema formation will contribute to adjusting current therapies and establishing new treatment strategies.

Aquaporins (AQPs) are a recently identified family of transmembrane water channel proteins that facilitate water transport and play a significant role in regulating water homeostasis across various tissues, including the nervous system (Zhou et al., 2022). Numerous recent studies have shown that AQP water channels are crucial for maintaining brain water balance and for the development and resolution of edema. This review specifically examines the role of AQPs in brain water transport and cerebral edema.

## 2 Aquaporins

### 2.1 Family members

**Figure 1:**
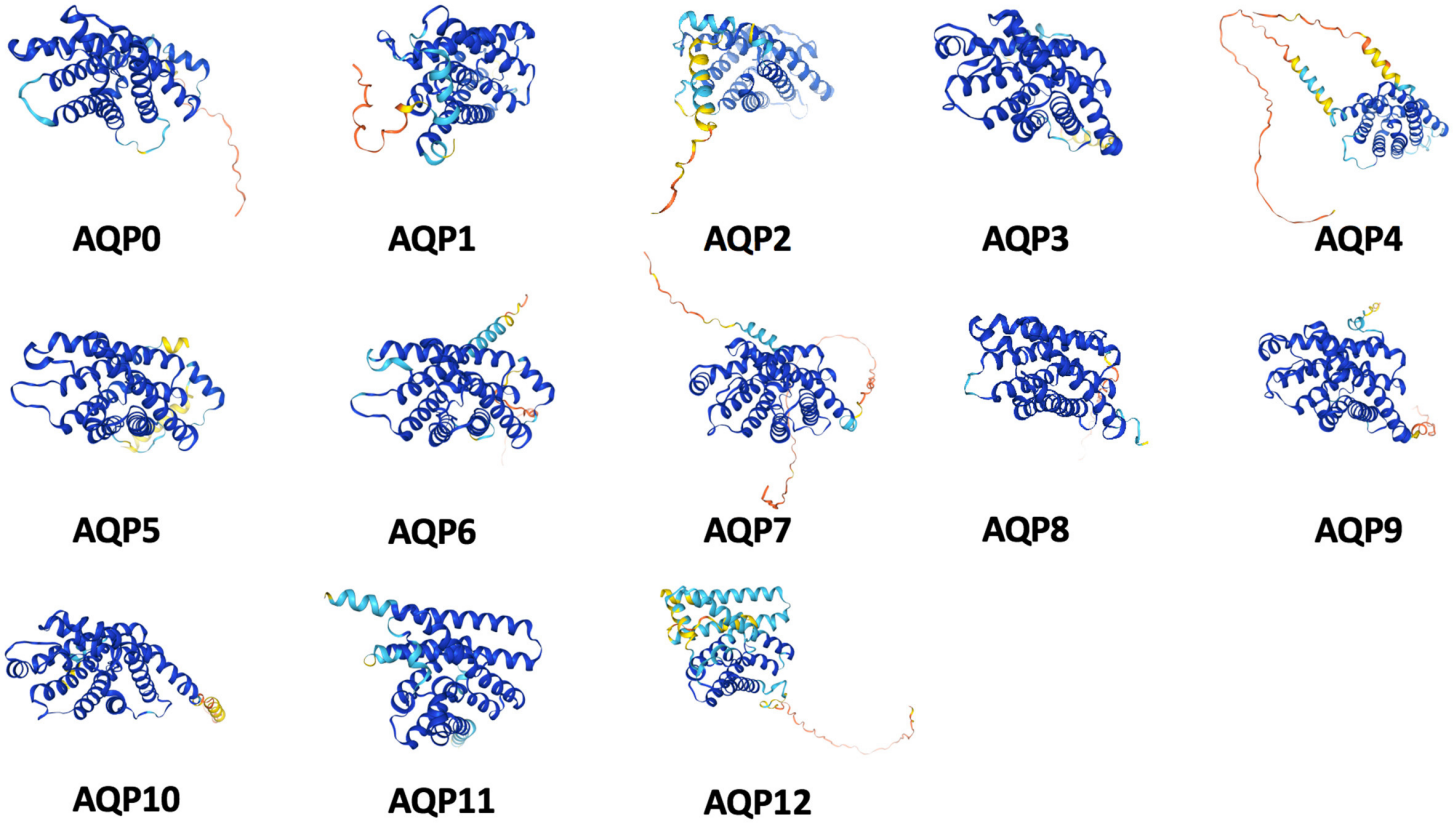
Schematic architectures of 13 isoforms (AQP0-12) in the AQP superfamily.

**Figure 2:**
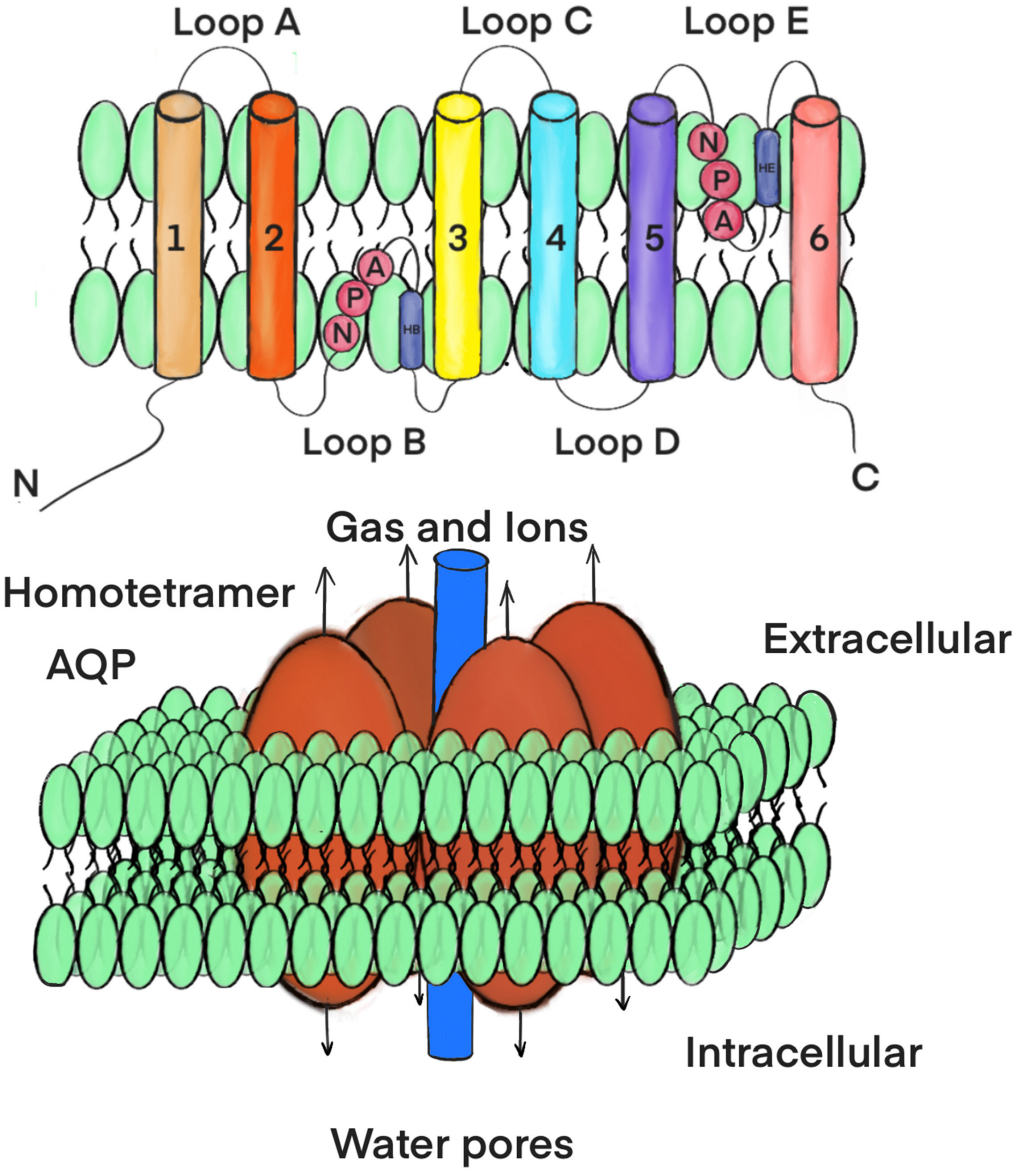
A secondary structure and topology of AQP molecule. AQP monomer has six membranespanning regions (1–6), five loops (A-E) with intracellular amino and carboxy termini as well as internal tandem repeats **(top)**. Each monomer has a water pore **(bottom)**.

To date, a total of 13 isoforms (AQP0–12) (Figure 1) are included in the AQP superfamily across numerous mammalian tissues, such as the kidney, nervous system, lung, liver, gastrointestinal tract, eye, heart, skin, and adipose tissue (Czyżewski et al., 2024). AQP monomers weigh approximately 28 kDa and typically encompass six highly hydrophobic transmembrane spanning domains and a central water pore (Eriksson et al., 2013) (Figure 2). These proteins are currently classified into three principal subfamilies based on their pore selectivity and sequence homology (da Silva et al., 2022): (a) conventional water-selective AQPs or aquaporins (AQP0,−1,−2,−4,−5,−6, and−8) are primarily permeable to water at a high flow rate. Nevertheless, AQP1, AQP6, and AQP8 also transport volatile solutes such as CO_2_, anions, and ammonia, respectively (Galli et al., 2021); (b) aquaglyceroporins (AQP3,−7,−9, and−10) are permeable to glycerol, urea, and other small solutes in addition to water; and (c) superaquaporins or subcellular aquaporins (AQP11 and 12) are localized within the cytosol and are involved in intracellular homeostasis. Additionally, a fourth subgroup was reported relatively recently, which included paralogs belonging to the aforementioned three subgroups (AQP0, 1, 3, 5, 8, 9, and 11), named peroxiporins. These AQPs have high permeability to hydrogen peroxide (H_2_O_2_) and play a significant role in eliminating excessive reactive oxygen species (ROS) (Varadaraj and Kumari, 2020; Montiel et al., 2020; Silva et al., 2022; Krüger et al., 2021; Zhang et al., 2022; Sorrentino et al., 2022). Table 1 summarizes the subdivision of the mammalian AQPs and their basic properties.

**Table 1:** Summary of mammalian AQPs and their basic properties.

**Gene symbol**	**Gene description**	**Synonym**	**Subfamily**	**Tissue expression**	**Permeability**
AQP0	Aquaporin 0	MIP	Classical aquaporins, peroxiporins	Eye lens (Chepelinsky, 2009)	H_2_O (Gorin et al., 1984), anions (Ehring et al., 1990), H_2_O_2_ (Varadaraj and Kumari, 2020)
AQP1	Aquaporin 1	CHIP28	Classical aquaporins, peroxiporins	Brain (Qiu et al., 2014), blood vessels (Mints et al., 2007), kidney proximal tubules (Seyahian et al., 2020), lung (Wang et al., 2022), digestive tracts (Liao et al., 2020), eye (Bogner et al., 2016), and ear (Huang et al., 2011)	H_2_O (Benga et al., 1986), CO_2_ (Galli et al., 2021), H_2_O_2_ (Mints et al., 2007)
AQP2	Aquaporin 2	NDI2, WCH-CD	Classical aquaporins	Kidney collecting ducts (Takata et al., 2022), trigeminal ganglia (Borsani et al., 2009)	H_2_O (Fushimi et al., 1993)
AQP3	Aquaporin 3	GIL	Aquglyceroporins, peroxiporins	Kidney collecting ducts (Lei et al., 2017), skin (Zhang et al., 2021), respiratory (Liu et al., 2007), and digestive tracts (Liao et al., 2020), and brain (Yang et al., 2011)	H_2_O, urea, glycerol (Ishibashi et al., 1994), NH_3_ (Litman et al., 2009), arsenite (Sosa et al., 2020), H_2_O_2_ (Silva et al., 2022)
AQP4	Aquaporin 4	MIWC, WCH4	Classical aquaporins	Brain (Skauli et al., 2022), lung (Wu et al., 2015), eye (Kimball et al., 2021), ear (Hirt et al., 2011), skeletal muscle (Chung et al., 2020), stomach parietal cells (Fukuhara et al., 2014), and kidney collecting ducts (Nielsen et al., 2002)	H_2_O (Jung et al., 1994), CO_2_ (Musa-Aziz et al., 2009), NH_3_ (Assentoft et al., 2016)
AQP5	Aquaporin 5	PPKB	Classical aquaporins, peroxiporins	Salivary, lacrimal, and sweat glands (Muroi and Isohama, 2021), brain (Yang et al., 2011), ear (Merves et al., 2003) and eye (Bogner et al., 2016)	H_2_O (Villandre et al., 2022), CO_2_ (Alishahi and Kamali, 2019), H_2_O_2_ (Silva et al., 2022)
AQP6	Aquaporin 6	KID	Classical aquaporins	Kidney collecting duct cells (Ohshiro et al., 2001) and brain (Nagase et al., 2007)	H_2_O (Molinas et al., 2017), urea, glycerol (Holm et al., 2004), NH_3_ (Musa-Aziz et al., 2009), anions (Galli et al., 2021)
AQP7	Aquaporin 7	GLYCQTL	Aquglyceroporins	Adipocytes (Iena et al., 2022), breast (Dai et al., 2020), testis (Saito et al., 2004), kidney, skeletal muscle (Iena and Lebeck, 2018), and brain (Shin et al., 2006)	H_2_O, urea, glycerol (Delporte et al., 2009), NH_3_ (Musa-Aziz et al., 2009), arsenite (Liu et al., 2002)
AQP8	Aquaporin 8	–	Classical aquaporins, peroxiporins	Pancreas, testis, liver (Ikaga et al., 2015), kidney (Molinas et al., 2012), heart (Yang et al., 2005), and brain (Yang et al., 2011)	H_2_O, NH_3_, urea (Kirscht et al., 2018), H_2_O_2_ (Krüger et al., 2021)
AQP9	Aquaporin 9	SSC1	Aquglyceroporins, peroxiporins	Liver (da Silva et al., 2022), leukocytes (Matsushima et al., 2014), testis (Arena et al., 2011) and brain (Yang et al., 2011)	H_2_O, urea, glycerol (Sugiyama et al., 2014), NH_3_ (Litman et al., 2009), arsenite (Liu et al., 2002), lactate (Medina et al., 2021), H_2_O_2_ (Zhang et al., 2022)
AQP10	Aquaporin 10	AQPA-HUMAN	Aquglyceroporins	Small intestine (Öberg et al., 2011) and testis (Hermo et al., 2004)	H_2_O, urea, glycerol (Ishibashi et al., 2002)
AQP11	Aquaporin 11	AQPX1	Superaquaporins, peroxiporins	Intestine (Zhu et al., 2016), liver, kidney (Ishibashi et al., 2014), testis (Shannonhouse et al., 2014), brain (Trillo-Contreras et al., 2022), heart (Verkerk et al., 2019), and adipose tissue (Frühbeck et al., 2020)	H_2_O, glycerol, H_2_O_2_ (Sorrentino et al., 2022; Frühbeck et al., 2020)
AQP12	Aquaporin 12	mAQP12	Superaquaporins	Pancreas (Ohta et al., 2009)	Undetermined

### 2.2 Presence in the brain

**Figure 3:**
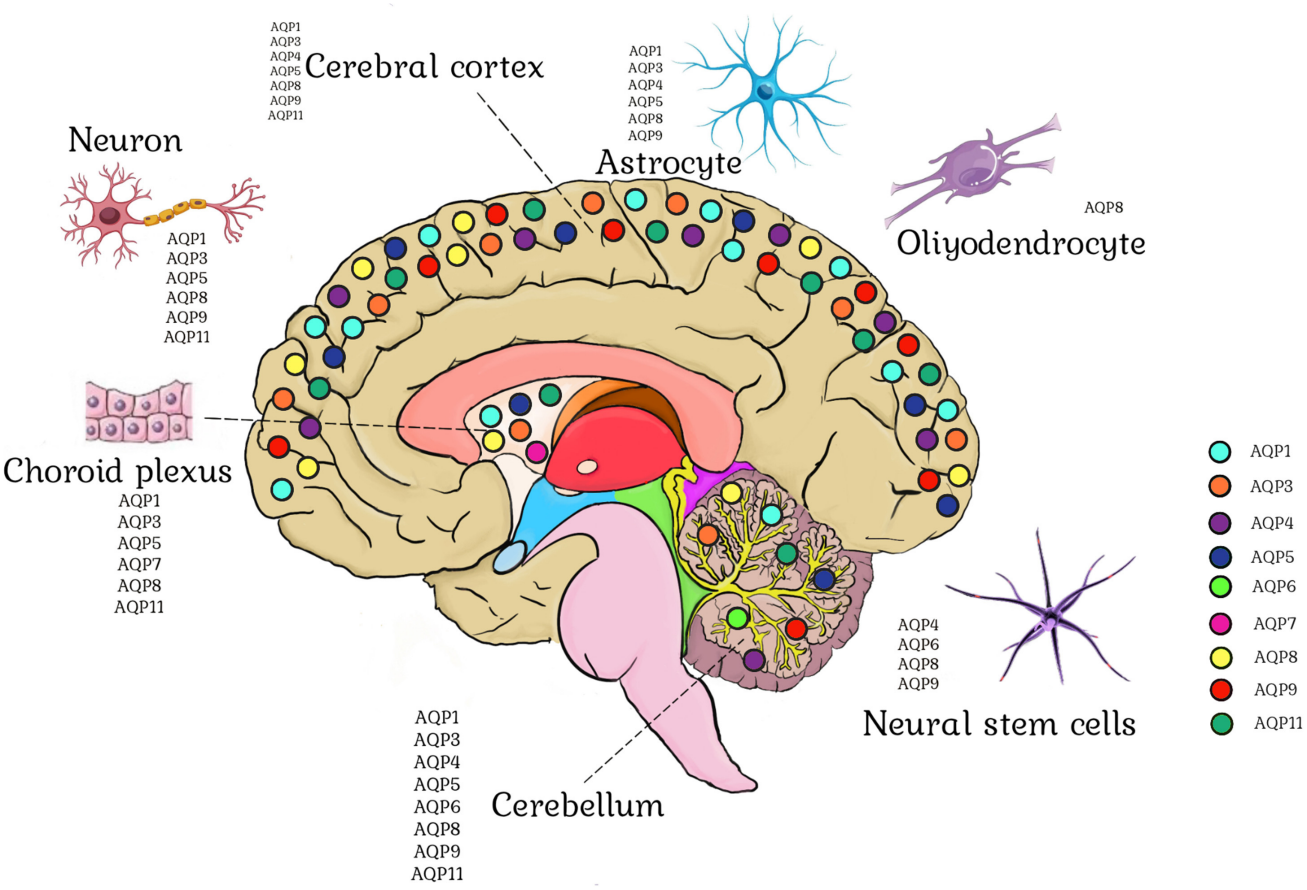
Brain aquaporin expression. Astrocytes express AQP1, AQP3, AQP4, AQP5, AQP8, and AQP9. Oligodendrocytes express AQP8. Neuron express AQP1, AQP3, AQP5, AQP8, AQP9, and AQP11. Neural stem cells express AQP4, AQP6, AQP8, and AQP9. AQP1, AQP3, AQP5, AQP7, AQP8, and AQP11 are expressed in the choroid plexus. AQP1, AQP3, AQP4, AQP5, AQP6, AQP8, AQP9, and AQP11 are found in the cerebellum. AQP1, AQP3, AQP4, AQP5, AQP8, AQP9, and AQP11 are expressed in the cerebral cortex.

To date, nine subtypes of AQP (AQP1, AQP3, AQP4, AQP5, AQP6, AQP7, AQP8, AQP9, and AQP11) have been recognized in various types of brain-resident cells, such as the cerebral cortex, cerebellum, choroid plexus, brain cells, and neural stem cells (as illustrated in Figure 3 and Table 2). However, it is important to note that only AQP1, AQP4, and AQP9 possess significant physiological functions and pathological implications. AQP4 is the most predominant and well-characterized aquaporin identified in the brain, with the brain being the primary site of AQP4 expression in mammals (Jung et al., 1994). In brain tissue, AQP4 is predominantly expressed in glia cells, particularly at the interface between the brain and major water-containing compartments (Rash et al., 1998), indicating its role in the movement of water into and out of the brain. AQP1 is expressed in the ventricular-facing cell plasma membrane of epithelial cells in the choroid plexus, suggesting a role of AQP1 in CSF secretion (Nielsen et al., 1993). AQP9 is the sole aquaglyceroporin expressed in astrocytes of the glia limitans and white matter tracts (Badaut et al., 2004). The function of AQP9 in the brain remains unclear, but its permeability to glycerol suggests that its likely involvement in brain metabolism. Although the transcriptional abundances of AQP1 and AQP9 are extremely low in human and mouse brain cells under physiological conditions, certain upregulations may occur under pathological circumstances (Misawa et al., 2008; Geistlinger et al., 2022).

**Table 2:** Expressions of AQPs in brain.

**AQP subfamilies**	**Expressions in brain**
AQP1	Cerebral cortex (Park et al., 2021), cerebellum (Wiegman et al., 2008), choroid plexus (Srisook et al., 2022), astrocyte (Sadashima et al., 2020), and neuron (Yu et al., 2020)
AQP3	Cerebral cortex (Yang et al., 2011), cerebellum (Yang et al., 2011), choroid plexus (Mobasheri et al., 2005), astrocyte (Yang et al., 2011), and neuron (Mobasheri et al., 2005)
AQP4	Cerebral cortex (Elsherbini et al., 2022), cerebellum (Zhao et al., 2020), astrocyte (Eide, 2022), and neural stem cells (Kong et al., 2008)
AQP5	Cerebral cortex (Antequera et al., 2022), cerebellum (Yang et al., 2011), choroid plexus (Sveinsdottir et al., 2014), astrocyte (Chai et al., 2013), and neuron (Chai et al., 2013)
AQP6	Cerebellum (Nagase et al., 2007) and neural stem cells (Lee et al., 2010)
AQP7	Choroid plexus (Shin et al., 2006)
AQP8	Cerebral cortex (Yamamoto et al., 2001), cerebellum, choroid plexus (Yang et al., 2011), astrocyte, oligodendrocyte, neuron (Yamamoto et al., 2001) and neural stem cells (La Porta et al., 2006)
AQP9	Cerebral cortex (Liu et al., 2018), cerebellum (Wiegman et al., 2008), astrocyte (Hirt et al., 2018), neuron (Mori et al., 2020) and neural stem cells (Cavazzin et al., 2006)
AQP11	Cerebral cortex, cerebellum (Gorelick et al., 2006), choroid plexus (Koike et al., 2016), and neuron (Gorelick et al., 2006)

Expressions of AQPs are based on detection of either mRNA or protein.

## 3 Role of AQPs in the brain water homeostasis

In the typical adult brain, water migrates between various compartments (CSF, blood, and both intracellular and interstitial brain parenchyma) in response to differences in hydrostatic and osmotic pressure. Water enters the brain through the blood-brain barrier (BBB) or the choroid plexus and exits through the arachnoid granulations into the venous system (Abbott, 2004). The significant expression of AQP4 at the BBB and in ependymal cells lining the ventricles has promoted initial studies on the crucial role of AQP4 in determining the water permeability of the BBB. AQP4 knockout mice exhibited greater ventricular enlargement and elevated ICP compared to wild-type mice in an experimental model of obstructive hydrocephalus, suggesting a function for AQP4 in CSF absorption (Bloch et al., 2006). Transgenic mice overexpressing AQP4 demonstrated an accelerated progression of brain swelling and poorer outcomes in water intoxication (Yang et al., 2008a). The deletion of AQP1 reduces osmotic water permeability in the choroid plexus by a factor of five. However, CSF production is only reduced by approximately 25% in AQP1 knockout mice compared to wild-type mice, indicating that only a portion of CSF secretion is AQP1-dependent and that there is a substantial contribution from extrachoroidal fluid production by the brain parenchyma (Oshio et al., 2005). Moreover, a study involving AQP1-deficient and AQP4-deficient mice concluded that AQP4, rather than AQP1, aids in mediating water flow into CSF, suggesting that AQP4 plays a vital role in CSF production (Igarashi et al., 2014). These phenotypic studies of AQP4 and AQP1 transgenic mice offer evidence for the roles of these water channels in the physiology of brain water homeostasis under normal and abnormal circumstances.

## 4 Role of AQPs in the brain edema

Klatzo classified edema into cytotoxic and vasogenic types (Klatzo, 1967). Fishman later added another type, termed interstitial (or hydrocephalic) edema, observed in patients with hydrocephalus (Fishman, 1975). Cytotoxic edema represents the intracellular accumulation of water reliant on ionic imbalances and affects glial, neuronal, and endothelial cells within the brain. The typical cytotoxic edema occurs in the early stages of ischemia or hypoxia, cerebral malaria, and hyponatremia, when brain cells are impaired but the BBB remains intact. In contrast, vasogenic edema emerges due to the disruption of the BBB, leading to an enhanced permeability of capillary endothelial cells to albumin and other plasma proteins (Hu et al., 2023). Nevertheless, in most instances, these two types of edema coexist, and one type of edema gradually transforms into the other (Pasantes-Morales and Cruz-Rangel, 2010). Interstitial cerebral edema refers to the outflow of CSF from the ventricles into the interstitial space of the brain. Patients with hydrocephalus or meningitis are affected by this etiology. The elevated pressure on the brain and CSF forces fluid into the brain parenchyma. The accumulation of fluid in the extracellular space, primarily in the white matter, leads to cerebral edema.

### 4.1 AQPs and cytotoxic edema

**Figure 4:**
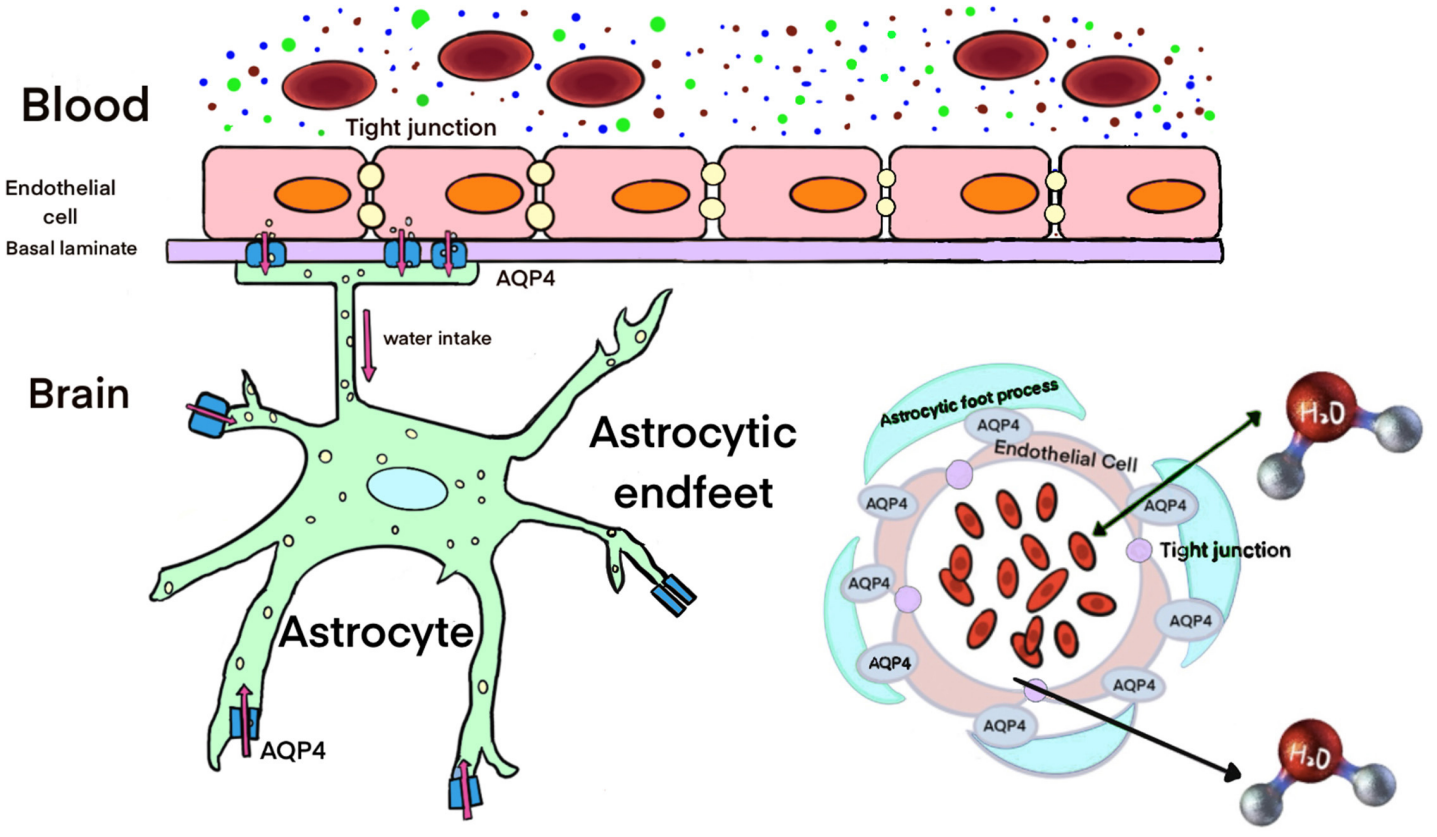
Role of AQP4 in cytotoxic edema. The swelling of the endfeet of astrocytes after water enters through the AQP4 water channel is a characteristic of cytotoxic edema.

Since astrocytes are the major cell type that swells in cytotoxic brain edema (Kimelberg, 1995) and are the predominant sites of AQP4 expression in the brain (González-Marrero et al., 2022), it can be logically inferred that AQP4 plays a crucial role in the development of cytotoxic brain edema (Figure 4). Manley et al. (2000) demonstrated that AQP4-deficient mice performed better than their wild-type counterparts in cytotoxic brain edema, employing two models of cytotoxic edema, namely acute water intoxication and early cerebral ischemia. Additionally, Papadopoulos and Verkman (2005) reported that AQP4-deficient mice had significantly less brain water accumulation in meningitis-induced brain edema, another cytotoxic edema model. Recently, Sucha et al. (2022) discovered that simultaneous deletion of AQP4 and Transient Receptor Potential Vanilloid 4 mitigated the cytotoxic edema following ischemic injury. AQP4 molecules are linked to the cell membrane by α-syntrophin, dystrophin, and other proteins, which are crucial for the localization of AQP4 to the astrocytic end-feet. Comparable studies have validated the role of AQP4 in cytotoxic edema using the dystrophin null *mdx-*β*geo* transgenic mice and the α-syntrophin null mice, which are considered as alternative models for the AQP4-null genotype (Moëlo et al., 2023; Vajda et al., 2002). Furthermore, AQP5 is also implicated in cytotoxic brain edema. Yamamoto et al. (2001) indicated that hypoxia elicited a significant decrease in AQP5 mRNA in rat astrocytes, suggesting that AQP5 might be one of the candidates for inducing cytotoxic edema in the central nervous system after ischemic injury.

### 4.2 AQPs and vasogenic edema

**Figure 5:**
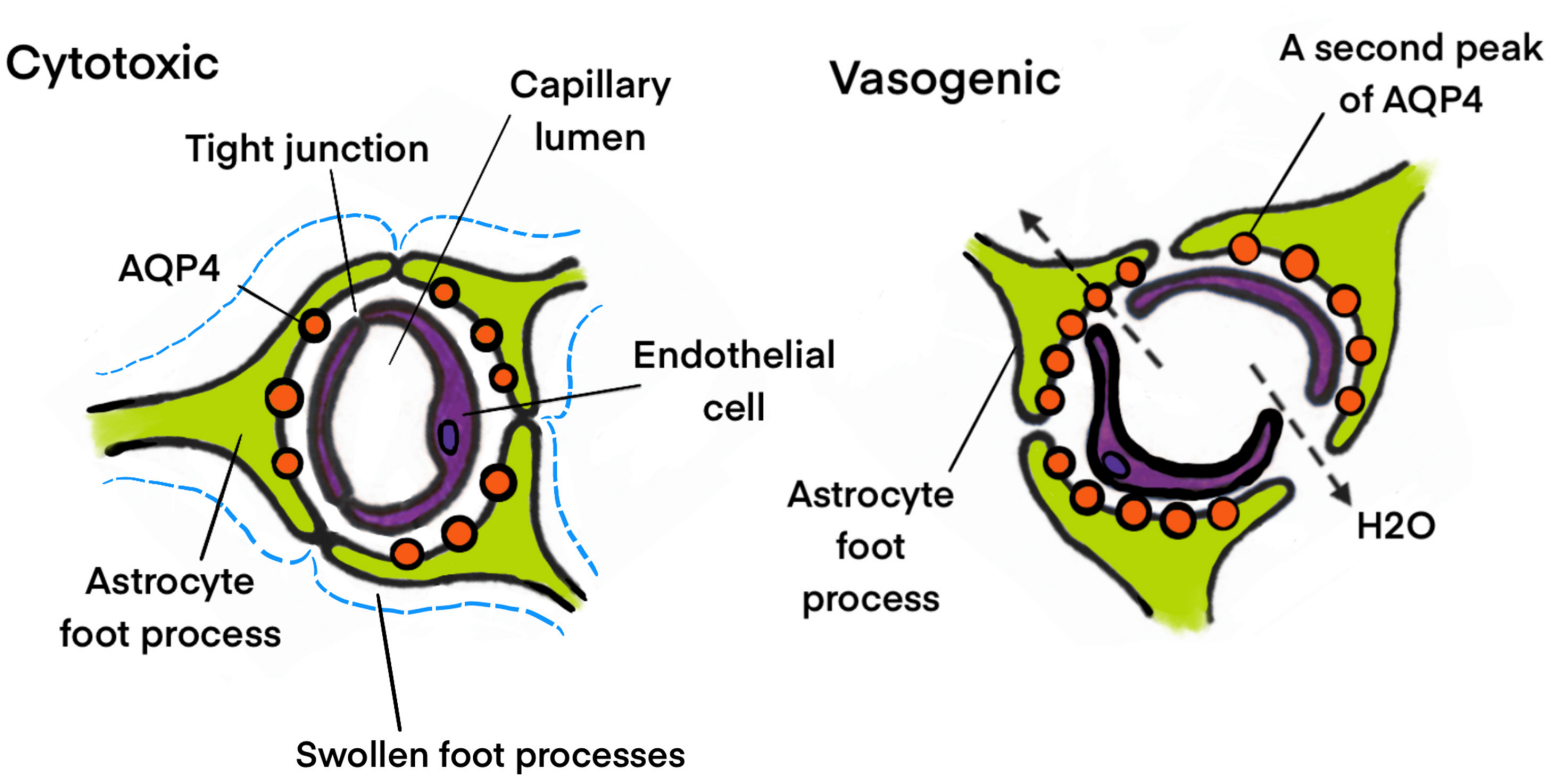
Different role of AQP4 in cytotoxic and vasogenic edema. In cytotoxic edema the water accumulation in astrocyte is AQP4 dependent. Contrary to cytotoxic edema, AQP4 plays a favorable role in water elimination in vasogenic edema.

AQP4 may not only facilitate water entry into the brain but also its exit (Figure 5). Overexpression of AQP4 has been observed in meningiomas, which is associated with the response to vasogenic edema of meningiomas (Faropoulos et al., 2021). In relation to vasogenic edema, Papadopoulos et al. (2004) noted that AQP4 deletion had the opposite effect (increased brain swelling) in three vasogenic edema models: intraparenchymal fluid infusion, focal cortical freeze injury, and brain tumor cell implantation. Following continuous intracerebral fluid infusion, AQP4-deficient mice exhibited higher ICP and brain water content compared to wild-type controls. Cortical freeze injury gives rise to a significantly greater brain water content and higher ICP in AQP4-deficient mice. In a brain tumor edema model involving stereotactic implantation of melanoma cells, AQP4-deficient mice showed higher ICP and accelerated neurological deterioration (Papadopoulos et al., 2004). A similar outcome was observed with the injection of *Staphylococcus aureus* to generate brain abscesses in the striatum of mice. AQP4-deficient mice exhibited more than twice the increase in brain water content, along with significantly higher ICP than wild-type control mice (Bloch et al., 2005). These findings suggest that AQP4 activators might alleviate vasogenic brain edema in humans. Additionally, AQP5 also plays a role in water homeostasis in vasogenic brain edema. Lambertz et al. (2013) demonstrated that the occurrence and severity of peritumoral edema in meningiomas were associated with AQP5 polymorphism A(-1364)C.

### 4.3 AQPs and interstitial (or hydrocephalic) edema

Given the crucial roles of AQP1 in CSF production and AQP4 in CSF absorption, it is proposed that they are involved in hydrocephalic brain edema resulting from the increase in CSF pressure and disruption of the BBB. The increased expression of AQP1 highlights the importance of excessive CSF production and an intact blood-CSF barrier during the development of hydrocephalic edema (Kim and Jung, 2011). Experiments have demonstrated that AQP1-null mice exhibit a fivefold reduction in osmotic water permeability in the choroid plexus compared to wild-type mice, indicating that reducing the function of AQP1 decreases the occurrence of non-obstructive hydrocephalus (Oshio et al., 2003). Several studies have reported elevated expression of AQP4 in connection with hydrocephalus, including congenital hydrocephalus in Texas rats (Paul et al., 2011), idiopathic communicating internal hydrocephalus in dogs (Schmidt et al., 2016), and inflammatory communicating hydrocephalus in rats (Tourdias et al., 2009). In patients with congenital hydrocephalus, the expression of AQP4 is elevated in both CSF and brain parenchyma (Castañeyra-Ruiz et al., 2013). In a model of obstructive hydrocephalus induced by kaolin injection, AQP4-deficient mice exhibited significant ventriculomegaly and increased ICP, which was suggested to result from impaired transependymal water clearance (Bloch et al., 2006). These discoveries suggest a compensatory function of AQPs in the water dynamics of hydrocephalic edema.

### 4.4 Regulation of AQPs expression in the brain edema

#### 4.4.1 Vasopressin

Vasopressin (VP) plays a crucial role in the development of brain injury and has been shown to regulate AQP4 expression in cases of brain edema. Studies indicate that astrocyte AQP4 levels were reduced by vasopressin 1a receptor (V1AR) antagonist treatment in a model of TBI, concurrently with the prevention of cell swelling and a reduction in edema (Marmarou et al., 2014; Taya et al., 2008). Additionally, V1AR has been found to upregulated cerebreal AQP1 expression, suggesting that V1AR may worsen brain edema formation post-TBI (Rauen et al., 2020). There is a substantial evidence suggesting that V1AR antagonists help reduce the development of cytotoxic brain edema by decreasing the upregulation of AQP4 (Kleindienst et al., 2013). In a stroke model induced by middle cerebral artery occlusion followed by reperfusion, it was observed that administering a V1AR antagonist decreased AQP4 expression in cytotoxic brain edema (Okuno et al., 2008).

#### 4.4.2 Dopamine

Dopamine has been demonstrated to curtail the proliferation of striatal astrocytes *in vitro* and downregulate AQP4 expression in primary cultured astrocytes (Küppers et al., 2008). It has been reported that AQP4 expression is augmented in the substantia nigra following tissue-type plasminogen activator-induced degeneration of dopaminergic neurons (Villarán et al., 2009). Collectively, these studies imply that dopamine might exert a persistent suppressive effect on AQP4 expression. In a model of TBI, it has been reported that dopamine deteriorates cerebral edema formation shortly after impact (Beaumont et al., 2000). This effect has been attributed to the vasopressor action of dopamine, which facilitated vasogenic edema.

#### 4.4.3 Erythropoietin

In recent years, a potent neuroprotective agent, erythropoietin (EPO), has been demonstrated to enhance the permeability of astrocyte AQP4 and might directly lower the risk of astrocyte swelling in stroke and other brain disorders (Gunnarson et al., 2009). In an animal hydrocephalus model, EPO treatment significantly mitigated the dilation of the cerebral ventricles in obstructive hydrocephalus by augmenting the expression of AQP4 (Rizwan Siddiqui et al., 2018). The administration of EPO post-TBI inhibited the reduction of AQP4, alleviated early cytotoxic brain edema, and maintained the structural and functional characteristics of the BBB, thereby weakening the vasogenic edema response (Blixt et al., 2018).

#### 4.4.4 Thyroid transcription factor-1

Thyroid transcription factor-1 (TTF-1), a transcriptional regulator containing a homeodomain and is coexpressed with AQP1 in the rat brain choroid plexus, has been reported to enhance the transcription of the AQP1 gene (Kim et al., 2007). Consequently, inhibiting the synthesis of TTF-1 increased the survival rate of animals with acute water intoxication-induced brain edema by suppressing AQP1 expression.

### 4.5 Interactions between AQP4 and other trans-membrane structures

#### 4.5.1 AQP4 and Kir4.1

Astrocytes-mediated potassium (K^+^) homeostasis is critically important for regulating neuronal excitability. An early study revealed that AQP4 is co-localized with the inward rectifier potassium channel Kir4.1 in the end-feet of retinal Müller cells, suggesting a functional interaction between the two (Nagelhus et al., 1999). The uncoupled expression of AQP4 and Kir4.1 on astrocytic end-feet contributes to the development of cytotoxic edema in the brain following subarachnoid hemorrhage (Yan et al., 2012).

#### 4.5.2 AQP4 and calcium signal transduction

Calcium (Ca^2+^) signaling mediates of bidirectional interactions between neurons and astrocytes. Impaired Ca^2+^ signaling plays a critical role in the progression of brain edema. Recent evidence suggests that AQP4 is involved in Ca^2+^ signaling in astrocytes. The deletion of AQP4 reduces Ca^2+^ signaling induced by hypo-osmotic stress in these cells (Thrane et al., 2011). This finding indicates that Ca^2+^ signals may result from AQP4-induced astrocyte swelling rather than being directly induced by AQP4 itself. Edema leads to astrocyte swelling, and the presence of AQP4 exacerbates this condition.

#### 4.5.3 AQP4 and Connexin 43

Connexin 43 (Cx43) is a prominent gap junction protein that is extensively expressed in the end-feet of astrocytes. It mediates intracellular interactions by facilitating the transport of ionic and molecules. Recent studies have linked Cx43 to abnormal neuro-differentiation associated with brain injury (Samarasinghe et al., 2014). Both Cx43 and AQP4 play crucial roles in the development of brain edema, with Cx43 potentially acting as a downstream effector of AQP4 (Li et al., 2015). The coordinated action of Cx43 and AQP4, along with K^+^ channels (Kir4.1/Kir5.1), ensures the efficient clearance of K^+^ from astrocytes into the vascular system (Lichter-Konecki et al., 2008).

### 4.6 AQPs and the glymphatic system in the brain edema

The glymphatic system (GS) is a recently discovered microscopic fluid clearance system composed of astrocytic perivascular tunnels that eliminate metabolic wastes from the brain. In this system, the CSF and interstitial fluid (ISF) continuously exchange. It comprises three key compartments: a periarterial CSF influx pathway, a perivenous ISF efflux pathway, and a parenchymal exchange pathway that relies on astrocytic AQP4 (Iliff et al., 2012). Since this discovery, increasing evidence indicates that the GS dysfunction is associated with the formation of brain edema, as it involves AQP4, which plays a crucial role in regulating water homeostasis. The water flow through AQP4 is bidirectional. AQP4 might potentially contribute to astrocytic swelling and cytotoxic edema by facilitating water influx into astrocytes during the early stages of ischemic stroke. However, in the later stages of ischemic stroke, when vasogenic edema becomes the primary cause of brain swelling due to the BBB breakdown, AQP4 could serve the purpose of alleviating brain edema by mediating the transport of excess water from the interstitial space to the GS. By promoting the AQP4 polarization to improve the GS function, it might facilitate the inflow of edema fluids, but it can also significantly enhance downstream drainage function, thereby reducing brain edema (Zhu et al., 2024).

## 5 Anti-edema drug: AQPs as targets

The management of brain edema involves sedation and the prevention of hypercapnia to avoid elevated intracranial pressure. This includes the administration of intravenous hyperosmolar solutions, such as mannitol and hypertonic saline. Corticosteroids are utilized for treating brain tumors. Surgical resection of the underlying lesion may be performed, and in severe cases, decompressive craniectomy is indicated. For critically ill patients, invasive monitoring of intracranial pressure and cerebral perfusion pressure is conducted to optimize treatment. However, many of these therapies for reducing brain swelling were introduced in the early to mid-20^th^ century, and their efficacy remains limited.

Water transport is a crucial process that contributes to human cerebral edema. Consequently, the utilization of AQP-targeted drugs to regulate water transport would offer novel prospects for therapeutic approaches to brain edema. Nevertheless, at present, there are currently a limited number of AQP-specific pharmacological modulators. Despite being non-specific, certain drugs that enhance or suppress AQP4 activity provide effective therapy in both the formation and resolution of edema. Bumetanide has been demonstrated to prevent edema formation following brain ischemia by reducing AQP4 expression (Migliati et al., 2010; Chen et al., 2023). However, bumetanide is a loop diuretic that inhibits the Na^+^/K^+^/2Cl^−^ cotransporter isoform (Boyarko et al., 2023), and its benefits on cerebral edema might also be attributed to the aberration of Na^+^/K^+^/2Cl^−^ cotransporter 1 expression in endothelial cells (Yang et al., 2019; O'Donnell et al., 2004). Therefore, it is challenging to comprehend the specific impact of AQP4 on edema formation. Acetazolamide (AZA), one of the most prevalently used drugs for lowering ICP and reducing CSF secretion, was also regarded as an inhibitor of AQP1 and AQP4 (Uldall et al., 2017; Gao et al., 2006). However, contrary to the aforementioned results, Yang et al. (2008b) found no significant inhibition of AQP4 water permeability by AZA. Similarly, methazolamide (MZA), another sulfonamide carbonic anhydrase inhibitor, also exhibited no significant effect on water permeability (Tanimura et al., 2009). Instead of a specific pharmacological drug to suppress AQP4, small interfering RNA (siRNA) technology has been employed to silence AQP4 in primary cultured astrocytes to prevent glutamate-induced astrocyte swelling (Lu et al., 2022). AQP4-siRNA was used to mitigate TBI induced edema by modifying post-traumatic AQP4 polarity reversal in rats (Lu et al., 2020). Similar findings have been documented by Guan et al. (2020), Chen et al. (2016), Fukuda et al. (2013), and others. While the siRNA approach is unlikely to be utilized as a specific drug for preventing edema formation, research on AQP4 inhibition by siRNA in animals will be valuable for validating the hypothesis that inhibiting AQP4 activity is a potential therapeutic target for managing brain edema.

## 6 Summary and future perspectives

In this review, data from cellular and *in vivo* animal studies strongly support the idea that AQPs play a functional role in brain water transport. The modulation of water transport is an integral component of controlling edema. Increasing evidence indicates that AQP4 has been shown to influence the accumulation and elimination of edema fluid. The careful modulation of AQP4 in different types of edema is particularly significant because AQP4 has dual roles in brain edema. In the early cytotoxic edema phase, AQP4 promotes the formation of edema fluid. In vasogenic brain edema, AQP4 enhances the rate of edema fluid elimination. Therefore, AQP4 inhibitors would be necessary to protect the brain in cytotoxic edema, while AQP4 activators are anticipated to facilitate the elimination of vasogenic brain edema. Unfortunately, as of now, no effective drugs for altering AQP4 expression or function have been identified. Additionally, AQP4 is expressed in many human tissues involved in crucial cellular and organ functions, such as urinary concentration, exocrine glandular secretion, and metabolism. Therefore, AQP4-selective therapeutic is required, which is a challenge since there are conserved amino acid sequences in the pore regions of various AQP subtypes. Furthermore, there are other brain-AQPs (AQP1 and 9) whose expression changes in brain disorders, but little is known about their pathophysiological effects. It is not clear whether there is an interaction between the different subtypes during edema and whether the other AQPs present in the brain also play roles in brain edema.

## References

[B1] Abbott N. J. (2004). Evidence for bulk flow of brain interstitial fluid: significance for physiology and pathology. Neurochem. Int. 45, 545–552. 10.1016/j.neuint.2003.11.006 15186921

[B2] Alishahi M. Kamali R. (2019). A novel molecular dynamics study of CO2 permeation through aquaporin-5. Eur. Phys. J. E Soft Matter. 42:151. 10.1140/epje/i2019-11912-x 31773315

[B3] Antequera D. Carrero L. Cunha Alves V. Ferrer I. Hernández-Gallego J. Municio C.. (2022). Differentially aquaporin 5 expression in submandibular glands and cerebral cortex in Alzheimer's disease. Biomedicines 10:1645. 10.3390/biomedicines10071645 35884950 PMC9312791

[B4] Arena S. Arena F. Maisano D. Di Benedetto V. Romeo C. Nicòtina P. A.. (2011). Aquaporin-9 immunohistochemistry in varicocele testes as a consequence of hypoxia in the sperm production site. Andrologia 43, 34–37. 10.1111/j.1439-0272.2009.01009.x 21219380

[B5] Assentoft M. Kaptan S. Schneider H. P. Deitmer J. W. de Groot B. L. MacAulay N.. (2016). Aquaporin 4 as a NH3 channel. J. Biol. Chem. 291, 19184–19195. 10.1074/jbc.M116.740217 27435677 PMC5009286

[B6] Badaut J. Petit J. M. Brunet J. F. Magistretti P. J. Charriaut-Marlangue C. Regli L.. (2004). Distribution of Aquaporin 9 in the adult rat brain: preferential expression in catecholaminergic neurons and in glial cells. Neuroscience 128, 27–38. 10.1016/j.neuroscience.2004.05.042 15450351

[B7] Beaumont A. Hayasaki K. Marmarou A. Barzo P. Fatouros P. Corwin F.. (2000). The effects of dopamine on edema formation in two models of traumatic brain injury. Acta Neurochir. Suppl. 76, 147–151. 10.1007/978-3-7091-6346-7_30 11449995

[B8] Benga G. Popescu O. Borza V. Pop V. I. Muresan A. Mocsy I.. (1986). Water permeability in human erythrocytes: identification of membrane proteins involved in water transport. Eur. J. Cell Biol. 41, 252–262. 3019699

[B9] Blixt J. Gunnarson E. Wanecek M. (2018). Erythropoietin attenuates the brain edema response after experimental traumatic brain injury. J. Neurotrauma. 35, 671–680. 10.1089/neu.2017.5015 29179621 PMC5806078

[B10] Bloch O. Auguste K. I. Manley G. T. Verkman A. S. (2006). Accelerated progression of kaolin-induced hydrocephalus in aquaporin-4-deficient mice. J. Cereb. Blood Flow Metab. 26, 1527–1537. 10.1038/sj.jcbfm.9600306 16552421

[B11] Bloch O. Papadopoulos M. C. Manley G. T. Verkman A. S. (2005). Aquaporin-4 gene deletion in mice increases focal edema associated with staphylococcal brain abscess. J. Neurochem. 95, 254–262. 10.1111/j.1471-4159.2005.03362.x 16181429

[B12] Bogner B. Schroedl F. Trost A. Kaser-Eichberger A. Runge C. Strohmaier C.. (2016). Aquaporin expression and localization in the rabbit eye. Exp. Eye Res. 147, 20–30. 10.1016/j.exer.2016.04.013 27107794

[B13] Borsani E. Bernardi S. Albertini R. Rezzani R. Rodella L. F. (2009). Alterations of AQP2 expression in trigeminal ganglia in a murine inflammation model. Neurosci. Lett. 449, 183–188. 10.1016/j.neulet.2008.11.014 19014999

[B14] Boyarko B. Podvin S. Greenberg B. Momper J. D. Huang Y. Gerwick W. H.. (2023). Evaluation of bumetanide as a potential therapeutic agent for Alzheimer's disease. Front. Pharmacol. 14:1190402. 10.3389/fphar.2023.1190402 37601062 PMC10436590

[B15] Castañeyra-Ruiz L. González-Marrero I. González-Toledo J. M. Castañeyra-Ruiz A. de Paz-Carmona H. Castañeyra-Perdomo A.. (2013). Aquaporin-4 expression in the cerebrospinal fluid in congenital human hydrocephalus. Fluids Barriers CNS 10:18. 10.1186/2045-8118-10-18 23659378 PMC3651869

[B16] Cavazzin C. Ferrari D. Facchetti F. Russignan A. Vescovi A. L. La Porta C. A.. (2006). Unique expression and localization of aquaporin-4 and aquaporin-9 in murine and human neural stem cells and in their glial progeny. Glia 53, 167–181. 10.1002/glia.20256 16206164

[B17] Chai R. C. Jiang J. H. Wong A. Y. Jiang F. Gao K. Vatcher G.. (2013). AQP5 is differentially regulated in astrocytes during metabolic and traumatic injuries. Glia 61, 1748–1765. 10.1002/glia.22555 23922257

[B18] Chen C. Fan P. Zhang L. Xue K. Hu J. Huang J.. (2023). Bumetanide rescues aquaporin-4 depolarization via suppressing β-dystroglycan cleavage and provides neuroprotection in rat retinal ischemia-reperfusion injury. Neuroscience 510, 95–108. 10.1016/j.neuroscience.2022.11.033 36493910

[B19] Chen J. Q. Zhang C. C. Jiang S. N. Lu H. Wang W. (2016). Effects of aquaporin 4 knockdown on brain edema of the uninjured side after traumatic brain injury in rats. Med. Sci. Monit. 22, 4809–4819. 10.12659/MSM.898190 27930615 PMC5161431

[B20] Chen X. Józsa T. I. Cardim D. Robba C. Czosnyka M. Payne S. J.. (2024). Modelling midline shift and ventricle collapse in cerebral oedema following acute ischaemic stroke. PLoS Comput. Biol. 20:e1012145. 10.1371/journal.pcbi.1012145 38805558 PMC11161059

[B21] Chepelinsky A. B. (2009). Structural function of MIP/aquaporin 0 in the eye lens; genetic defects lead to congenital inherited cataracts. Handb. Exp. Pharmacol. 190, 265–297. 10.1007/978-3-540-79885-9_14 19096783

[B22] Chung S. W. Kim J. Y. Yoon J. P. Suh D. W. Yeo W. J. Lee Y. S.. (2020). Atrogin1-induced loss of aquaporin 4 in myocytes leads to skeletal muscle atrophy. Sci. Rep. 10:14189. 10.1038/s41598-020-71167-8 32843684 PMC7447774

[B23] Czyżewski W. Litak J. Sobstyl J. Mandat T. Torres K. Staśkiewicz G.. (2024). Aquaporins: gatekeepers of fluid dynamics in traumatic brain injury. Int. J. Mol. Sci. 25:6553. 10.3390/ijms25126553 38928258 PMC11204105

[B24] da Silva I. V. Garra S. Calamita G. Soveral G. (2022). The multifaceted role of aquaporin-9 in health and its potential as a clinical biomarker. Biomolecules 12:897. 10.3390/biom12070897 35883453 PMC9313442

[B25] Dai C. Charlestin V. Wang M. Walker Z. T. Miranda-Vergara M. C. Facchine B. A.. (2020). Aquaporin-7 regulates the response to cellular stress in breast cancer. Cancer Res. 80, 4071–4086. 10.1158/0008-5472.CAN-19-2269 32631905 PMC7899076

[B26] Delporte C. Virreira M. Crutzen R. Louchami K. Sener A. Malaisse W. J.. (2009). Functional role of aquaglyceroporin 7 expression in the pancreatic beta-cell line BRIN-BD11. J. Cell. Physiol. 221, 424–429. 10.1002/jcp.21872 19585522

[B27] Ehring G. R. Zampighi G. Horwitz J. Bok D. Hall J. E. (1990). Properties of channels reconstituted from the major intrinsic protein of lens fiber membranes. J. Gen. Physiol. 96, 631–664. 10.1085/jgp.96.3.631 1700061 PMC2229003

[B28] Eide P. K. (2022). Cellular changes at the glia-neuro-vascular interface in definite idiopathic normal pressure hydrocephalus. Front. Cell. Neurosci. 16:981399. 10.3389/fncel.2022.981399 36119130 PMC9478415

[B29] Elsherbini D. M. A. Ghoneim F. M. El-Mancy E. M. Ebrahim H. A. El-Sherbiny M. El-Shafey M.. (2022). Astrocytes profiling in acute hepatic encephalopathy: Possible enrolling of glial fibrillary acidic protein, tumor necrosis factor-alpha, inwardly rectifying potassium channel (Kir 4.1) and aquaporin-4 in rat cerebral cortex. Front. Cell. Neurosci. 16:896172. 10.3389/fncel.2022.896172 36060277 PMC9428715

[B30] Eriksson U. K. Fischer G. Friemann R. Enkavi G. Tajkhorshid E. Neutze R.. (2013). Subangstrom resolution X-ray structure details aquaporin-water interactions. Science 340, 1346–1349. 10.1126/science.1234306 23766328 PMC4066176

[B31] Faropoulos K. Polia A. Tsakona C. Pitaraki E. Moutafidi A. Gatzounis G.. (2021). Evaluation of AQP4/TRPV4 channel co-expression, microvessel density, and its association with peritumoral brain edema in intracranial meningiomas. J. Mol. Neurosci. 71, 1786–1795. 10.1007/s12031-021-01801-1 33538957 PMC8799549

[B32] Fishman R. A. (1975). Brain edema. N. Engl. J. Med. 293, 706–711. 10.1056/NEJM197510022931407 1160939

[B33] Frühbeck G. Balaguer I. Méndez-Giménez L. Valent,í V. Becerril S. Catalán V.. (2020). Aquaporin-11 contributes to TGF-β1-induced endoplasmic reticulum stress in human visceral adipocytes: role in obesity-associated inflammation. Cells 9:1403. 10.3390/cells9061403 32512939 PMC7349025

[B34] Fukuda A. M. Adami A. Pop V. Bellone J. A. Coats J. S. Hartman R. E.. (2013). Posttraumatic reduction of edema with aquaporin-4 RNA interference improves acute and chronic functional recovery. J. Cereb. Blood Flow Metab. 33, 1621–1632. 10.1038/jcbfm.2013.118 23899928 PMC3790933

[B35] Fukuhara S. Matsuzaki J. Tsugawa H. Masaoka T. Miyoshi S. Mori H.. (2014). Mucosal expression of aquaporin-4 in the stomach of histamine type 2 receptor knockout mice and Helicobacter pylori-infected mice. J. Gastroenterol. Hepatol. 29, 53–59. 10.1111/jgh.12771 25521734

[B36] Fushimi K. Uchida S. Hara Y. Hirata Y. Marumo F. Sasaki S.. (1993). Cloning and expression of apical membrane water channel of rat kidney collecting tubule. Nature 361, 549–552. 10.1038/361549a0 8429910

[B37] Galli M. Hameed A. Żbikowski A. Zabielski P. (2021). Aquaporins in insulin resistance and diabetes: more than channels! *Redox Biol*. 44:102027. 10.1016/j.redox.2021.102027 34090243 PMC8182305

[B38] Gao J. Wang X. Chang Y. Zhang J. Song Q. Yu H.. (2006). osmotic water permeability by interaction with aquaporin-1. Anal. Biochem. 350, 165–170. 10.1016/j.ab.2006.01.003 16480680

[B39] Geistlinger K. Schmidt J. D. R. Beitz E. (2022). Lactic acid permeability of aquaporin-9 enables cytoplasmic lactate accumulation via an ion trap. Life 12:120. 10.3390/life12010120 35054513 PMC8779662

[B40] González-Marrero I. Hernández-Abad L. G. González-Gómez M. Soto-Viera M. Carmona-Calero E. M. Castañeyra-Ruiz L.. (2022). Altered expression of AQP1 and AQP4 in brain barriers and cerebrospinal fluid may affect cerebral water balance during chronic hypertension. Int. J. Mol. Sci. 23:12277. 10.3390/ijms232012277 36293145 PMC9603298

[B41] Gorelick D. A. Praetorius J. Tsunenari T. Nielsen S. Agre P. (2006). Aquaporin-11: a channel protein lacking apparent transport function expressed in brain. BMC Biochem. 7:14. 10.1186/1471-2091-7-14 16650285 PMC1475587

[B42] Gorin M. B. Yancey S. B. Cline J. Revel J. P. Horwitz J. (1984). The major intrinsic protein (MIP) of the bovine lens fiber membrane: characterization and structure based on cDNA cloning. Cell 39, 49–59. 10.1016/0092-8674(84)90190-9 6207938

[B43] Guan Y. Li L. Chen J. Lu H. (2020). Effect of AQP4-RNAi in treating traumatic brain edema: Multi-modal MRI and histopathological changes of early stage edema in a rat model. Exp. Ther. Med. 19, 2029–2036. 10.3892/etm.2020.8456 32104262 PMC7027281

[B44] Gunnarson E. Song Y. Kowalewski J. M. Brismar H. Brines M. Cerami A.. (2009). Erythropoietin modulation of astrocyte water permeability as a component of neuroprotection. Proc. Natl. Acad. Sci. USA. 106, 1602–1607. 10.1073/pnas.0812708106 19164545 PMC2629445

[B45] Hermo L. Krzeczunowicz D. Ruz R. (2004). Cell specificity of aquaporins 0, 3, and 10 expressed in the testis, efferent ducts, and epididymis of adult rats. J. Androl. 25, 494–505. 10.1002/j.1939-4640.2004.tb02820.x 15223838

[B46] Hirt B. Gleiser C. Eckhard A. Mack A. F. Müller M. Wolburg H.. (2011). All functional aquaporin-4 isoforms are expressed in the rat cochlea and contribute to the formation of orthogonal arrays of particles. Neuroscience 189, 79–92. 10.1016/j.neuroscience.2011.05.037 21621589

[B47] Hirt L. Price M. Mastour N. Brunet J. F. Barrière G. Friscourt F.. (2018). Increase of aquaporin 9 expression in astrocytes participates in astrogliosis. J. Neurosci. Res. 96, 194–206. 10.1002/jnr.24061 28419510

[B48] Holm L. M. Klaerke D. A. Zeuthen T. (2004). Aquaporin 6 is permeable to glycerol and urea. Pflugers Arch. 448, 181–186. 10.1007/s00424-004-1245-x 14985982

[B49] Hu S. Exner C. Sienel R. I. Wehn A. Seker B. Magdane Boldoczki F.. (2023). Characterization of vasogenic and cytotoxic brain edema formation after experimental TBI by free water diffusion MRI. J. Neurotrauma. 41, 393–406. 10.1089/neu.2023.0222 37776177 PMC10908318

[B50] Huang Y. D. Xia S. W. Dai P. Han D. Y. (2011). Role of AQP1 in inner ear in motion sickness. Physiol. Behav. 104, 749–753. 10.1016/j.physbeh.2011.07.031 21839760

[B51] Iena F. M. Kalucka J. Nielsen L. Søndergaard E. Nielsen S. Lebeck J.. (2022). Localization of aquaglyceroporins in human and murine white adipose tissue. Histochem. Cell Biol. 157, 623–639. 10.1007/s00418-022-02090-4 35235046

[B52] Iena F. M. Lebeck J. (2018). Implications of Aquaglyceroporin 7 in Energy Metabolism. Int. J. Mol. Sci. 19:154. 10.3390/ijms19010154 29300344 PMC5796103

[B53] Igarashi H. Tsujita M. Kwee I. L. Nakada T. (2014). Water influx into cerebrospinal fluid is primarily controlled by aquaporin-4, not by aquaporin-1: 17O JJVCPE MRI study in knockout mice. Neuroreport 25, 39–43. 10.1097/WNR.0000000000000042 24231830 PMC4235386

[B54] Ikaga R. Namekata I. Kotiadis V. N. Ogawa H. Duchen M. R. Tanaka H.. (2015). Knockdown of aquaporin-8 induces mitochondrial dysfunction in 3T3-L1 cells. Biochem. Biophys. Rep. 4, 187–195. 10.1016/j.bbrep.2015.09.009 29124204 PMC5668916

[B55] Iliff J. J. Wang M. Liao Y. Plogg B. A. Peng W. Gundersen G. A.. (2012). A paravascular pathway facilitates CSF flow through the brain parenchyma and the clearance of interstitial solutes, including amyloid β. Sci. Transl. Med. 4:147ra.111. 10.1126/scitranslmed.3003748 22896675 PMC3551275

[B56] Ishibashi K. Morinaga T. Kuwahara M. Sasaki S. Imai M. (2002). Cloning and identification of a new member of water channel (AQP10) as an aquaglyceroporin. Biochim. Biophys. Acta. 1576, 335–340. 10.1016/S0167-4781(02)00393-7 12084581

[B57] Ishibashi K. Sasaki S. Fushimi K. Uchida S. Kuwahara M. Saito H.. (1994). Molecular cloning and expression of a member of the aquaporin family with permeability to glycerol and urea in addition to water expressed at the basolateral membrane of kidney collecting duct cells. Proc. Natl. Acad. Sci. USA. 91, 6269–6273. 10.1073/pnas.91.14.6269 7517548 PMC44182

[B58] Ishibashi K. Tanaka Y. Morishita Y. (2014). The role of mammalian superaquaporins inside the cell. Biochim. Biophys. Acta. 1840, 1507–1512. 10.1016/j.bbagen.2013.10.039 24189537

[B59] Jung J. S. Bhat R. V. Preston G. M. Guggino W. B. Baraban J. M. Agre P.. (1994). Molecular characterization of an aquaporin cDNA from brain: candidate osmoreceptor and regulator of water balance. Proc. Natl. Acad. Sci. USA. 91, 13052–13056. 10.1073/pnas.91.26.13052 7528931 PMC45579

[B60] Kim J. Jung Y. (2011). Different expressions of AQP1, AQP4, eNOS, and VEGF proteins in ischemic versus non-ischemic cerebropathy in rats: potential roles of AQP1 and eNOS in hydrocephalic and vasogenic edema formation. Anat. Cell Biol. 44, 295–303. 10.5115/acb.2011.44.4.295 22254158 PMC3254883

[B61] Kim J. G. Son Y. J. Yun C. H. Kim Y. I. Nam-Goong I. S. Park J. H.. (2007). Thyroid transcription factor-1 facilitates cerebrospinal fluid formation by regulating aquaporin-1 synthesis in the brain. J. Biol. Chem. 282, 14923–14931. 10.1074/jbc.M701411200 17371871

[B62] Kimball E. Schaub J. Quillen S. Keuthan C. Pease M. E. Korneva A.. (2021). The role of aquaporin-4 in optic nerve head astrocytes in experimental glaucoma. PLoS ONE 16:e0244123. 10.1371/journal.pone.0244123 33529207 PMC7853498

[B63] Kimelberg H. K. (1995). Current concepts of brain edema: review of laboratory investigations. J. Neurosurg. 83, 1051–1059. 10.3171/jns.1995.83.6.1051 7490620

[B64] Kirscht A. Sonntag Y. Kjellbom P. Johanson U. (2018). A structural preview of aquaporin 8 via homology modeling of seven vertebrate isoforms. BMC Struct. Biol. 18:2. 10.1186/s12900-018-0081-8 29454339 PMC5816522

[B65] Klatzo I. (1967). Presidental address. Neuropathological aspects of brain edema. J. Neuropathol. Exp. Neurol. 26, 1–14. 10.1097/00005072-196701000-00001 5336776

[B66] Kleindienst A. Dunbar J. G. Glisson R. Marmarou A. (2013). The role of vasopressin V1A receptors in cytotoxic brain edema formation following brain injury. Acta Neurochir. 155, 151–164. 10.1007/s00701-012-1558-z 23188468

[B67] Koike S. Tanaka Y. Matsuzaki T. Morishita Y. Ishibashi K. (2016). Aquaporin-11 (AQP11) expression in the mouse brain. Int. J. Mol. Sci. 17:861. 10.3390/ijms17060861 27258268 PMC4926395

[B68] Kong H. Fan Y. Xie J. Ding J. Sha L. Shi X.. (2008). AQP4 knockout impairs proliferation, migration and neuronal differentiation of adult neural stem cells. J. Cell Sci. 121, 4029–4036. 10.1242/jcs.035758 19033383

[B69] Krüger C. Jörns A. Kaynert J. Waldeck-Weiermair M. Michel T. Elsner M.. (2021). The importance of aquaporin-8 for cytokine-mediated toxicity in rat insulin-producing cells. Free Radic. Biol. Med. 174, 135–143. 10.1016/j.freeradbiomed.2021.08.003 34363947

[B70] Küppers E. Gleiser C. Brito V. Wachter B. Pauly T. Hirt B.. (2008). AQP4 expression in striatal primary cultures is regulated by dopamine–implications for proliferation of astrocytes. Eur. J. Neurosci. 28, 2173–2182. 10.1111/j.1460-9568.2008.06531.x 19046364

[B71] La Porta C. A. Gena P. Gritti A. Fascio U. Svelto M. Calamita G.. (2006). Adult murine CNS stem cells express aquaporin channels. Biol. Cell 98, 89–94. 10.1042/BC20040153 15907198

[B72] Lambertz N. Hindy N. E. Adler C. Rump K. Adamzik M. Keyvani K.. (2013). Expression of aquaporin 5 and the AQP5 polymorphism A(-1364)C in association with peritumoral brain edema in meningioma patients. J. Neurooncol. 112, 297–305. 10.1007/s11060-013-1064-z 23392848

[B73] Lee J. S. Cho W. J. Shin L. Jena B. P. (2010). Involvement of cholesterol in synaptic vesicle swelling. Exp. Biol. Med. 235, 470–477. 10.1258/ebm.2010.009259 20407079

[B74] Lei L. Wang W. Jia Y. Su L. Zhou H. Verkman A. S.. (2017). Aquaporin-3 deletion in mice results in renal collecting duct abnormalities and worsens ischemia-reperfusion injury. Biochim. Biophys. Acta Mol. Basis Dis. 1863, 1231–1241. 10.1016/j.bbadis.2017.03.012 28344130

[B75] Li G. Liu X. Liu Z. Su Z. (2015). Interactions of connexin 43 and aquaporin-4 in the formation of glioma-induced brain edema. Mol. Med. Rep. 11, 1188–1194. 10.3892/mmr.2014.2867 25373717

[B76] Liao S. Gan L. Lv L. Mei Z. (2020). The regulatory roles of aquaporins in the digestive system. Genes Dis. 8, 250–258. 10.1016/j.gendis.2019.12.011 33997172 PMC8093583

[B77] Lichter-Konecki U. Mangin J. M. Gordish-Dressman H. Hoffman E. P. Gallo V. (2008). Gene expression profiling of astrocytes from hyperammonemic mice reveals altered pathways for water and potassium homeostasis in vivo. Glia 56, 365–377. 10.1002/glia.20624 18186079 PMC4116685

[B78] Litman T. Søgaard R. Zeuthen T. (2009). Ammonia and urea permeability of mammalian aquaporins. Handb. Exp. Pharmacol. 190, 327–358. 10.1007/978-3-540-79885-9_17 19096786

[B79] Liu J. Y. Chen X. X. Chen H. Y. Shi J. Leung G. P. Tang S. C.. (2018). Downregulation of aquaporin 9 exacerbates beta-amyloid-induced neurotoxicity in Alzheimer's disease models in vitro and in vivo. Neuroscience 394, 72–82. 10.1016/j.neuroscience.2018.09.016 30266683

[B80] Liu Y. L. Matsuzaki T. Nakazawa T. Murata S. Nakamura N. Kondo T.. (2007). Expression of aquaporin 3 (AQP3) in normal and neoplastic lung tissues. Hum. Pathol. 38, 171–178. 10.1016/j.humpath.2006.07.015 17056099

[B81] Liu Z. Shen J. Carbrey J. M. Mukhopadhyay R. Agre P. Rosen B. P.. (2002). Arsenite transport by mammalian aquaglyceroporins AQP7 and AQP9. Proc. Natl. Acad. Sci. USA. 99, 6053–6058. 10.1073/pnas.092131899 11972053 PMC122900

[B82] Lu H. Zhan Y. Ai L. Chen H. Chen J. (2020). AQP4-siRNA alleviates traumatic brain edema by altering post-traumatic AQP4 polarity reversal in TBI rats. J. Clin. Neurosci. 81, 113–119. 10.1016/j.jocn.2020.09.015 33222898

[B83] Lu Q. Xiong J. Yuan Y. Ruan Z. Zhang Y. Chai B.. (2022). Minocycline improves the functional recovery after traumatic brain injury via inhibition of aquaporin-4. Int. J. Biol. Sci. 18, 441–458. 10.7150/ijbs.64187 34975343 PMC8692149

[B84] Manley G. T. Fujimura M. Ma T. Noshita N. Filiz F. Bollen A. W.. (2000). Aquaporin-4 deletion in mice reduces brain edema after acute water intoxication and ischemic stroke. Nat. Med. 6, 159–163. 10.1038/72256 10655103

[B85] Marmarou C. R. Liang X. Abidi N. H. Parveen S. Taya K. Henderson S. C.. (2014). Selective vasopressin-1a receptor antagonist prevents brain edema, reduces astrocytic cell swelling and GFAP, V1aR and AQP4 expression after focal traumatic brain injury. Brain Res. 1581, 89–102. 10.1016/j.brainres.2014.06.005 24933327 PMC4240002

[B86] Matsushima A. Ogura H. Koh T. Shimazu T. Sugimoto H. (2014). Enhanced expression of aquaporin 9 in activated polymorphonuclear leukocytes in patients with systemic inflammatory response syndrome. Shock 42, 322–326. 10.1097/SHK.0000000000000218 24978896

[B87] Medina Y. Acosta L. Reppetti J. Corominas A. Bustamante J. Szpilbarg N.. (2021). Lactic acid transport mediated by aquaporin-9: implications on the pathophysiology of preeclampsia. Front. Physiol. 12:774095. 10.3389/fphys.2021.774095 34925067 PMC8678610

[B88] Merves M. Krane C. M. Dou H. Greinwald J. H. Menon A. G. Choo D.. (2003). Expression of aquaporin 1 and 5 in the developing mouse inner ear and audiovestibular assessment of an Aqp5 null mutant. J. Assoc. Res. Otolaryngol. 4, 264–275. 10.1007/s10162-002-3033-7 12943377 PMC3202717

[B89] Migliati E. R. Amiry-Moghaddam M. Froehner S. C. Adams M. E. Ottersen O. P. Bhardwaj A.. (2010). Na(+)-K (+)-2Cl (-) cotransport inhibitor attenuates cerebral edema following experimental stroke via the perivascular pool of aquaporin-4. Neurocrit. Care. 13, 123–131. 10.1007/s12028-010-9376-8 20458553

[B90] Mints M. Hildenbrand A. Lalitkumar L. P. Andersson S. Nielsen S. Gemzell-Danielsson K.. (2007). Expression of aquaporin-1 in endometrial blood vessels in menorrhagia. Int. J. Mol. Med. 19, 407–411. 10.3892/ijmm.19.3.407 17273788

[B91] Misawa T. Arima K. Mizusawa H. Satoh J. (2008). Close association of water channel AQP1 with amyloid-beta deposition in Alzheimer disease brains. Acta Neuropathol. 116, 247–260. 10.1007/s00401-008-0387-x 18509662 PMC2516196

[B92] Mobasheri A. Wray S. Marples D. (2005). Distribution of AQP2 and AQP3 water channels in human tissue microarrays. J. Mol. Histol. 36, 1–14. 10.1007/s10735-004-2633-4 15703994

[B93] Moëlo C. Quillévéré A. Le Roy L. Timsit S. (2023). (S)-roscovitine, a CDK inhibitor, decreases cerebral edema and modulates AQP4 and α1-syntrophin interaction on a pre-clinical model of acute ischemic stroke. Glia 72, 322–337. 10.1002/glia.24477 37828900

[B94] Molinas A. Turkina M. V. Magnusson K. E. Mirazimi A. Vikström E. (2017). Perturbation of wound healing, cytoskeletal organization and cellular protein networks during hazara virus infection. Front. Cell Dev. Biol. 5:98. 10.3389/fcell.2017.00098 29209610 PMC5702460

[B95] Molinas S. M. Trumper L. Marinelli R. A. (2012). Mitochondrial aquaporin-8 in renal proximal tubule cells: evidence for a role in the response to metabolic acidosis. Am. J. Physiol. Renal Physiol. 303, F458–F466. 10.1152/ajprenal.00226.2012 22622463

[B96] Montiel V. Bella R. Michel L. Y. M. Esfahani H. De Mulder D. Robinson E. L.. (2020). Inhibition of aquaporin-1 prevents myocardial remodeling by blocking the transmembrane transport of hydrogen peroxide. Sci. Transl. Med. 12:eaay2176. 10.1093/ehjci/ehaa946.3665 33028705

[B97] Mori S. Kurimoto T. Miki A. Maeda H. Kusuhara S. Nakamura M.. (2020). Aqp9 gene deletion enhances retinal ganglion cell (RGC) death and dysfunction induced by optic nerve crush: evidence that aquaporin 9 acts as an astrocyte-to-neuron lactate shuttle in concert with monocarboxylate transporters to support rgc function and survival. Mol. Neurobiol. 57, 4530–4548. 10.1007/s12035-020-02030-0 32748371 PMC7515957

[B98] Muroi S. I. Isohama Y. (2021). C-terminal domain of aquaporin-5 is required to pass its protein quality control and ensure its trafficking to plasma membrane. Int. J. Mol. Sci. 22:13461. 10.3390/ijms222413461 34948259 PMC8707437

[B99] Musa-Aziz R. Chen L. M. Pelletier M. F. Boron W. F. (2009). Relative CO2/NH3 selectivities of AQP1, AQP4, AQP5, AmtB, and RhAG. Proc. Natl. Acad. Sci. USA. 106, 5406–5411. 10.1073/pnas.0813231106 19273840 PMC2664022

[B100] Nagase H. Agren J. Saito A. Liu K. Agre P. Hazama A.. (2007). Molecular cloning and characterization of mouse aquaporin 6. Biochem. Biophys. Res. Commun. 352, 12–16. 10.1016/j.bbrc.2006.10.110 17112474 PMC2504719

[B101] Nagelhus E. A. Horio Y. Inanobe A. Fujita A. Haug F. M. Nielsen S.. (1999). Immunogold evidence suggests that coupling of K+ siphoning and water transport in rat retinal Müller cells is mediated by a coenrichment of Kir4.1 and AQP4 in specific membrane domains. Glia 26, 47–54. 10.1002/(sici)1098-1136(199903)26:1<47::aid-glia5>3.0.co;2-5 10088671

[B102] Nielsen S. Frøkiaer J. Marples D. Kwon T. H. Agre P. Knepper M. A.. (2002). Aquaporins in the kidney: from molecules to medicine. Physiol. Rev. 82, 205–244. 10.1152/physrev.00024.2001 11773613

[B103] Nielsen S. Smith B. L. Christensen E. I. Agre P. (1993). Distribution of the aquaporin CHIP in secretory and resorptive epithelia and capillary endothelia. Proc. Natl. Acad. Sci. USA. 90, 7275–7279. 10.1073/pnas.90.15.7275 8346245 PMC47119

[B104] Öberg F. Sjöhamn J. Fischer G. Moberg A. Pedersen A. Neutze R.. (2011). Glycosylation increases the thermostability of human aquaporin 10 protein. J. Biol. Chem. 286, 31915–31923. 10.1074/jbc.M111.242677 21733844 PMC3173105

[B105] O'Donnell M. E. Tran L. Lam T. I. Liu X. B. Anderson S. E. (2004). Bumetanide inhibition of the blood-brain barrier Na-K-Cl cotransporter reduces edema formation in the rat middle cerebral artery occlusion model of stroke. J. Cereb. Blood Flow Metab. 24, 1046–1056. 10.1097/01.WCB.0000130867.32663.90 15356425

[B106] Ohshiro K. Yaoita E. Yoshida Y. Fujinaka H. Matsuki A. Kamiie J.. (2001). Expression and immunolocalization of AQP6 in intercalated cells of the rat kidney collecting duct. Arch. Histol. Cytol. 64, 329–338. 10.1679/aohc.64.329 11575429

[B107] Ohta E. Itoh T. Nemoto T. Kumagai J. Ko S. B. Ishibashi K.. (2009). Pancreas-specific aquaporin 12 null mice showed increased susceptibility to caerulein-induced acute pancreatitis. Am. J. Physiol. Cell Physiol. 297, C1368–C1378. 10.1152/ajpcell.00117.2009 19726746

[B108] Okuno K. Taya K. Marmarou C. R. Ozisik P. Fazzina G. Kleindienst A.. (2008). The modulation of aquaporin-4 by using PKC-activator (phorbol myristate acetate) and V1a receptor antagonist (SR49059) following middle cerebral artery occlusion/reperfusion in the rat. Acta Neurochir. Suppl. 102, 431–436. 10.1007/978-3-211-85578-2_84 19388361

[B109] Oshio K. Song Y. Verkman A. S. Manley G. T. (2003). Aquaporin-1 deletion reduces osmotic water permeability and cerebrospinal fluid production. Acta Neurochir. Suppl. 86, 525–528. 10.1007/978-3-7091-0651-8_107 14753499

[B110] Oshio K. Watanabe H. Song Y. Verkman A. S. Manley G. T. (2005). Reduced cerebrospinal fluid production and intracranial pressure in mice lacking choroid plexus water channel Aquaporin-1. FASEB J. 19, 76–78. 10.1096/fj.04-1711fje 15533949

[B111] Papadopoulos M. C. Manley G. T. Krishna S. Verkman A. S. (2004). Aquaporin-4 facilitates reabsorption of excess fluid in vasogenic brain edema. FASEB J. 18, 1291–1293. 10.1096/fj.04-1723fje 15208268

[B112] Papadopoulos M. C. Verkman A. S. (2005). Aquaporin-4 gene disruption in mice reduces brain swelling and mortality in pneumococcal meningitis. J. Biol. Chem. 280, 13906–13912. 10.1074/jbc.M413627200 15695511

[B113] Park J. Madan M. Chigurupati S. Baek S. H. Cho Y. Mughal M. R.. (2021). Neuronal aquaporin 1 inhibits amyloidogenesis by suppressing the interaction between beta-secretase and amyloid precursor protein. J. Gerontol. A Biol. Sci. Med. Sci. 76, 23–31. 10.1093/gerona/glaa068 32154567 PMC7756701

[B114] Pasantes-Morales H. Cruz-Rangel S. (2010). Brain volume regulation: osmolytes and aquaporin perspectives. Neuroscience 168, 871–884. 10.1016/j.neuroscience.2009.11.074 20004708

[B115] Paul L. Madan M. Rammling M. Chigurupati S. Chan S. L. Pattisapu J. V.. (2011). Expression of aquaporin 1 and 4 in a congenital hydrocephalus rat model. Neurosurgery 68, 462–473. 10.1227/NEU.0b013e3182011860 21135737

[B116] Qiu B. Li X. Sun X. Wang Y. Jing Z. Zhang X.. (2014). Overexpression of aquaporin-1 aggravates hippocampal damage in mouse traumatic brain injury models. Mol. Med. Rep. 9, 916–922. 10.3892/mmr.2014.1899 24430824

[B117] Rash J. E. Yasumura T. Hudson C. S. Agre P. Nielsen S. (1998). Direct immunogold labeling of aquaporin-4 in square arrays of astrocyte and ependymocyte plasma membranes in rat brain and spinal cord. Proc. Natl. Acad. Sci. USA. 95, 11981–11986. 10.1073/pnas.95.20.11981 9751776 PMC21751

[B118] Rauen K. Pop V. Trabold R. Badaut J. Plesnila N. (2020). Vasopressin V1a receptors regulate cerebral aquaporin 1 after traumatic brain injury. J. Neurotrauma. 37, 665–674. 10.1089/neu.2019.6653 31547764 PMC7045352

[B119] Rizwan Siddiqui M. Attar F. Mohanty V. Kim K. S. Shekhar Mayanil C. Tomita T.. (2018). Erythropoietin-mediated activation of aquaporin-4 channel for the treatment of experimental hydrocephalus. Childs. Nerv. Syst. 34, 2195–2202. 10.1007/s00381-018-3865-z 29982881 PMC6208663

[B120] Sadashima S. Honda H. Suzuki S. O. Shijo M. Aishima S. Kai K.. (2020). Accumulation of astrocytic aquaporin 4 and aquaporin 1 in prion protein plaques. J. Neuropathol. Exp. Neurol. 79, 419–429. 10.1093/jnen/nlaa010 32167542

[B121] Saito K. Kageyama Y. Okada Y. Kawakami S. Kihara K. Ishibashi K.. (2004). Localization of aquaporin-7 in human testis and ejaculated sperm: possible involvement in maintenance of sperm quality. J. Urol. 172, 2073–2076. 10.1097/01.ju.0000141499.08650.ab 15540792

[B122] Samarasinghe R. A. Kanuparthi P. S. Timothy Greenamyre J. DeFranco D. B. Di Maio R. (2014). Transient muscarinic and glutamatergic stimulation of neural stem cells triggers acute and persistent changes in differentiation. Neurobiol. Dis. 70, 252–261. 10.1016/j.nbd.2014.06.020 25003306 PMC4152385

[B123] Schmidt M. J. Rummel C. Hauer J. Kolecka M. Ondreka N. McClure V.. (2016). Increased CSF aquaporin-4, and interleukin-6 levels in dogs with idiopathic communicating internal hydrocephalus and a decrease after ventriculo-peritoneal shunting. Fluids Barriers CNS. 13:12. 10.1186/s12987-016-0034-1 27357498 PMC4928270

[B124] Seyahian E. A. Cacciagiu L. Damiano A. E. Zotta E. (2020). AQP1 expression in the proximal tubule of diabetic rat kidney. Heliyon 6:e03192. 10.1016/j.heliyon.2020.e03192 31956716 PMC6956755

[B125] Shannonhouse J. L. Urbanski H. F. Woo S. L. Fong L. A. Goddard S. D. Lucas W. F.. (2014). Aquaporin-11 control of testicular fertility markers in Syrian hamsters. Mol. Cell. Endocrinol. 391, 1–9. 10.1016/j.mce.2014.04.011 24791736 PMC4368057

[B126] Shin I. Kim H. J. Lee J. E. Gye M. C. (2006). Aquaporin7 expression during perinatal development of mouse brain. Neurosci. Lett. 409, 106–111. 10.1016/j.neulet.2006.09.075 17052846

[B127] Silva P. M. da Silva I. V. Sarmento M. J. Silva Í. C. Carvalho F. A. Soveral G.. (2022). Aquaporin-3 and aquaporin-5 facilitate migration and cell-cell adhesion in pancreatic cancer by modulating cell biomechanical properties. Cells 11:1308. 10.3390/cells11081308 35455986 PMC9030499

[B128] Skauli N. Savchenko E. Ottersen O. P. Roybon L. Amiry-Moghaddam M. (2022). Canonical bone morphogenetic protein signaling regulates expression of aquaporin-4 and its anchoring complex in mouse astrocytes. Front. Cell. Neurosci. 16:878154. 10.3389/fncel.2022.878154 35518645 PMC9067306

[B129] Sorrentino I. Galli M. Medraño-Fernandez I. Sitia R. (2022). Transfer of H2O2 from Mitochondria to the endoplasmic reticulum via Aquaporin-11. Redox Biol. 55:102410. 10.1016/j.redox.2022.102410 35863264 PMC9304643

[B130] Sosa C. Guillén N. Lucea S. Sorribas V. (2020). Effects of oral exposure to arsenite on arsenic metabolism and transport in rat kidney. Toxicol. Lett. 333, 4–12. 10.1016/j.toxlet.2020.07.029 32736004

[B131] Srisook C. Glaharn S. Punsawad C. Viriyavejakul P. (2022). Apoptotic changes and aquaporin-1 expression in the choroid plexus of cerebral malaria patients. Malar. J. 21:43. 10.1186/s12936-022-04044-6 35151337 PMC8841049

[B132] Sucha P. Hermanova Z. Chmelova M. Kirdajova D. Camacho Garcia S. Marchetti V.. (2022). The absence of AQP4/TRPV4 complex substantially reduces acute cytotoxic edema following ischemic injury. Front. Cell. Neurosci. 16:1054919. 10.3389/fncel.2022.1054919 36568889 PMC9773096

[B133] Sugiyama Y. Yamazaki K. Kusaka-Kikushima A. Nakahigashi K. Hagiwara H. Miyachi Y.. (2014). Analysis of aquaporin 9 expression in human epidermis and cultured keratinocytes. FEBS Open Bio. 4, 611–616. 10.1016/j.fob.2014.06.004 25161869 PMC4141191

[B134] Sveinsdottir S. Gram M. Cinthio M. Sveinsdottir K. Mörgelin M. Ley D.. (2014). Altered expression of aquaporin 1 and 5 in the choroid plexus following preterm intraventricular hemorrhage. Dev. Neurosci. 36, 542–551. 10.1159/000366058 25342576

[B135] Takata T. Hamada S. Mae Y. Iyama T. Ogihara R. Seno M.. (2022). Uromodulin regulates murine aquaporin-2 activity via thick ascending limb-collecting duct cross-talk during water deprivation. Int. J. Mol. Sci. 23:9410. 10.3390/ijms23169410 36012675 PMC9408883

[B136] Tanimura Y. Hiroaki Y. Fujiyoshi Y. (2009). Acetazolamide reversibly inhibits water conduction by aquaporin-4. J. Struct. Biol. 166, 16–21. 10.1016/j.jsb.2008.11.010 19114109

[B137] Taya K. Gulsen S. Okuno K. Prieto R. Marmarou C. R. Marmarou A.. (2008). Modulation of AQP4 expression by the selective V1a receptor antagonist, SR49059, decreases trauma-induced brain edema. Acta Neurochir. Suppl. 102, 425–429. 10.1007/978-3-211-85578-2_83 19388360

[B138] Thrane A. S. Rappold P. M. Fujita T. Torres A. Bekar L. K. Takano T.. (2011). Critical role of aquaporin-4 (AQP4) in astrocytic Ca2+ signaling events elicited by cerebral edema. Proc. Natl. Acad. Sci. USA. 108, 846–851. 10.1073/pnas.1015217108 21187412 PMC3021020

[B139] Tourdias T. Dragonu I. Fushimi Y. Deloire M. S. Boiziau C. Brochet B.. (2009). Aquaporin 4 correlates with apparent diffusion coefficient and hydrocephalus severity in the rat brain: a combined MRI-histological study. Neuroimage 47, 659–666. 10.1016/j.neuroimage.2009.04.070 19409501

[B140] Trillo-Contreras J. L. Ramírez-Lorca R. Villadiego J. Echevarría M. (2022). Cellular distribution of brain aquaporins and their contribution to cerebrospinal fluid homeostasis and hydrocephalus. Biomolecules 12:530. 10.3390/biom12040530 35454119 PMC9025855

[B141] Uldall M. Botfield H. Jansen-Olesen I. Sinclair A. Jensen R. (2017). Acetazolamide lowers intracranial pressure and modulates the cerebrospinal fluid secretion pathway in healthy rats. Neurosci. Lett. 645, 33–39. 10.1016/j.neulet.2017.02.032 28219789

[B142] Vajda Z. Pedersen M. Füchtbauer E. M. Wertz K. Stødkilde-Jørgensen H. Sulyok E.. (2002). Delayed onset of brain edema and mislocalization of aquaporin-4 in dystrophin-null transgenic mice. Proc. Natl. Acad. Sci. USA. 99, 13131–13136. 10.1073/pnas.192457099 12232046 PMC130598

[B143] Varadaraj K. Kumari S. S. (2020). Lens aquaporins function as peroxiporins to facilitate membrane transport of hydrogen peroxide. Biochem. Biophys. Res. Commun. 524, 1025–1029. 10.1016/j.bbrc.2020.02.031 32063362 PMC7085977

[B144] Verkerk A. O. Lodder E. M. Wilders R. (2019). Aquaporin channels in the heart-physiology and pathophysiology. Int. J. Mol. Sci. 20:2039. 10.3390/ijms20082039 31027200 PMC6514906

[B145] Villandre J. White V. Lear T. B. Chen Y. Tuncer F. Vaiz E.. (2022). A repurposed drug screen for compounds regulating aquaporin 5 stability in lung epithelial cells. Front. Pharmacol. 13:828643. 10.3389/fphar.2022.828643 35145418 PMC8821664

[B146] Villarán R. F. de Pablos R. M. Argüelles S. Espinosa-Oliva A. M. Tomás-Camardiel M. Herrera A. J.. (2009). The intranigral injection of tissue plasminogen activator induced blood-brain barrier disruption, inflammatory process and degeneration of the dopaminergic system of the rat. Neurotoxicology 30, 403–413. 10.1016/j.neuro.2009.02.011 19442825

[B147] Wang Q. Li Y. Wu C. Wang T. Wu M. (2022). Aquaporin-1 inhibition exacerbates ischemia-reperfusion-induced lung injury in mice. Am. J. Med. Sci. 5:S0002. 10.1016/j.amjms.2022.08.017 36075463

[B148] Wiegman M. J. Bullinger L. V. Kohlmeyer M. M. Hunter T. C. Cipolla M. J. (2008). Regional expression of aquaporin 1, 4, and 9 in the brain during pregnancy. Reprod. Sci. 15, 506–516. 10.1177/1933719107311783 18579859

[B149] Wu Y. Pan C. Y. Guo C. Z. Dong Z. J. Wu Q. Dong H. M.. (2015). Expression of aquaporin 1 and 4 in rats with acute hypoxic lung injury and its significance. Genet. Mol. Res. 14, 12756–12764. 10.4238/2015.October.19.19 26505426

[B150] Yamamoto N. Yoneda K. Asai K. Sobue K. Tada T. Fujita Y.. (2001). Alterations in the expression of the AQP family in cultured rat astrocytes during hypoxia and reoxygenation. Brain Res. Mol. Brain Res. 90, 26–38. 10.1016/S0169-328X(01)00064-X 11376853

[B151] Yan J. H. Khatibi N. H. Han H. B. Hu Q. Chen C. H. Li L.. (2012). p53-induced uncoupling expression of aquaporin-4 and inwardly rectifying K+ 4.1 channels in cytotoxic edema after subarachnoid hemorrhage. CNS Neurosci. Ther. 18, 334–342. 10.1111/j.1755-5949.2012.00299.x 22420318 PMC6493666

[B152] Yang B. Song Y. Zhao D. Verkman A. S. (2005). Phenotype analysis of aquaporin-8 null mice. Am. J. Physiol. Cell Physiol. 288, C1161–C1170. 10.1152/ajpcell.00564.2004 15647389

[B153] Yang B. Zador Z. Verkman A. S. (2008a). Glial cell aquaporin-4 overexpression in transgenic mice accelerates cytotoxic brain swelling. J. Biol. Chem. 283, 15280–15286. 10.1074/jbc.M801425200 18375385 PMC2397463

[B154] Yang B. Zhang H. Verkman A. S. (2008b). Lack of aquaporin-4 water transport inhibition by antiepileptics and arylsulfonamides. Bioorg. Med. Chem. 16, 7489–7493. 10.1016/j.bmc.2008.06.005 18572411 PMC3325054

[B155] Yang M. Gao F. Liu H. Yu W. H. He G. Q. Zhuo F.. (2011). Immunolocalization of aquaporins in rat brain. Anat. Histol. Embryol. 40, 299–306. 10.1111/j.1439-0264.2011.01070.x 21496068

[B156] Yang X. L. Zeng M. L. Shao L. Jiang G. T. Cheng J. J. Chen T. X.. (2019). NFAT5 and HIF-1α coordinate to regulate NKCC1 expression in hippocampal neurons after hypoxia-ischemia. Front. Cell Dev. Biol. 7:339. 10.3389/fcell.2019.00339 31921851 PMC6923656

[B157] Yu B. Zhang J. Li H. Sun X. (2020). Silencing of aquaporin1 activates the Wnt signaling pathway to improve cognitive function in a mouse model of Alzheimer's disease. Gene 755:144904. 10.1016/j.gene.2020.144904 32540373

[B158] Zhang B. Lv D. Chen Y. Nie W. Jiao Y. Zhang J.. (2022). Aquaporin-9 facilitates liver regeneration following hepatectomy. Redox Biol. 50:102246. 10.1016/j.redox.2022.102246 35086002 PMC8802049

[B159] Zhang H. Cai W. Shao X. (2021). Regulation of aquaporin-3 water permeability by hyaluronan. Phys. Chem. Chem. Phys. 23, 25706–25711. 10.1039/D1CP02867G 34755729

[B160] Zhao L. Luo Z. Qiu S. Jia Y. Zhong S. Chen G.. (2020). Abnormalities of aquaporin-4 in the cerebellum in bipolar II disorder: an ultra-high b-values diffusion weighted imaging study. J. Affect. Disord. 274, 136–143. 10.1016/j.jad.2020.05.035 32469796

[B161] Zhou Z. Zhan J. Cai Q. Xu F. Chai R. Lam K.. (2022). The water transport system in astrocytes-aquaporins. Cells 11:2564. 10.3390/cells11162564 36010640 PMC9406552

[B162] Zhu C. Chen Z. Jiang Z. (2016). Expression, distribution and role of aquaporin water channels in human and animal stomach and intestines. Int. J. Mol. Sci. 17:1399. 10.3390/ijms17091399 27589719 PMC5037679

[B163] Zhu J. Mo J. Liu K. Chen Q. Li Z. He Y.. (2024). Glymphatic system impairment contributes to the formation of brain edema after ischemic stroke. Stroke 55, 1393–1404. 10.1161/STROKEAHA.123.045941 38533660

